# Overview of AI-Based Scent Creation

**DOI:** 10.3390/s26082568

**Published:** 2026-04-21

**Authors:** Takamichi Nakamoto, Manuel Aleixandre

**Affiliations:** Institute of Integrated Research (llR), Institute of Science Tokyo, Yokohama 226-0026, Japan; aleixandre.m.aa@m.titech.ac.jp

**Keywords:** scent creation, odor descriptor, natural language processing, deep neural network, mass spectrum, odor reproduction, olfactory display, optimization, diffusion model

## Abstract

Although odor classification and odor quantification by e-nose have been studied for a long time, the next stage is to express a detected scent using language. The methods used to map molecular structure parameters, mass spectra, and sensor responses onto language expression are reviewed first. NLP (Natural Language Processing) is useful for that purpose. Conversely, the linguistic expression of the scent can be transformed into sensing data. The odor mixture can be generated so that the measured response pattern can be identical to that of the scent to be created. Two methods, optimization-based and generative AI-based ones, to search for the recipe of the created scent, are explained. Finally, the intended odor is generated using an olfactory display. We provide the latest information on the emerging technology of scent creation.

## 1. Introduction

A perfumer can make the intended scent, although it is very difficult for laymen to do it. When two different scents are blended, it is usually hard to predict olfactory perception of the blended scent from each individual scent. However, a perfumer with rich experience can do it. Is it possible for digital technologies to create a novel intended scent? Perfume industries have developed AI-assisted systems for perfumers. Givodan’s Carto [1], IBM’s Philyra [2], and Fermenich’s Scentmate [3] are used to formulate new fragrances. Philyra by IBM uses AI to find unexplored combinations of chemical compounds. It uses a large database of fragrance formulas and consumer preferences. Scentmate by Ferminich has an AI-based platform which helps customers create and select perfumes so that development time can be reduced. However, they are based on proprietary data and methods not available for open academic research. Their main purpose is to assist perfumers in exploring combinations of ingredients, so their functions are limited. Although physical AI [4], which can perform actual tasks through a computer interface, is recently becoming popular, “Chemical AI”, which contributes to chemical perception, has so far not been studied much.

However, currently, new algorithmic approaches to create a scent from linguistic information are evolving. In addition to assisting perfumer’s work, it will become easy to attach the scents to digital content such as movies, games, etc. [5,6,7]. We cannot prepare the intended scents for digital content immediately. Moreover, it is not easy for a content creator to not make some scents commercially available. These algorithms for creating scents are useful since commercially available scents are very limited. People can enjoy scented digital content if any scent can be easily attached, although it is quite limited at the current stage. Automatic scent creation can contribute to spatial computing, entertainment computing, etc.

Before scent creation, several techniques are required. First, the sensing data should be predicted from odor perception data. Next, odor reproduction using a small set of odor components is necessary. Third, an olfactory display to present scents using odor components is required.

In Section 2, we introduce the studies to prepare for creating scents. Then, two algorithmic scent generation methods are explained, followed by discussion and conclusion.

## 2. Work Related to Scent Creation

### 2.1. Prediction Method of Odor Perception

#### 2.1.1. Odor-Descriptor Prediction Using Molecular Structure Parameters

The first step to create a scent is to predict the odor impression. When someone sniffs a scent, they express the impression of the perceived scent. We often use a set of odor descriptors corresponding to olfactory perception, such as “warm”, “fresh”, “floral”, “fruity”, etc. It is possible to predict odor descriptors using cheminformatic techniques.

Castro et al. studied odor-descriptor data to extract the information of odor categories [8]. Moreover, several researchers have studied the prediction method of the scent impression from molecular structure parameters. Several representative techniques to predict odor descriptors were explained here.

**Figure 1:**
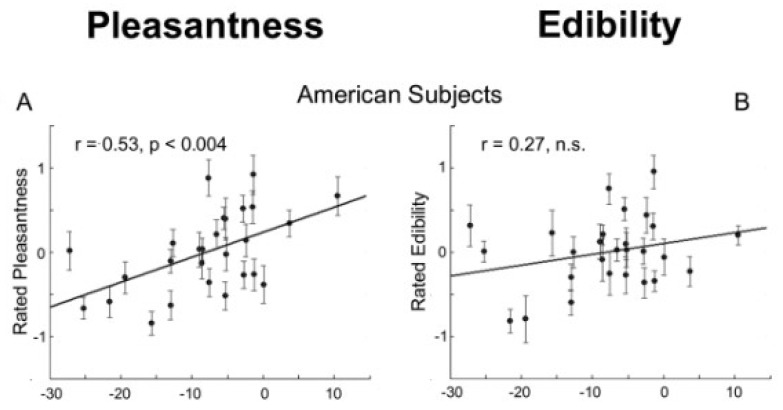
Result of odor impression prediction for unknown molecules, (**A**) pleasantness and (**B**) edibility vs. principal component 1 of the physicochemical parameters [10]. n.s. = not significant.

Many researchers have used the molecular structure parameters, often generated by Dragon software, for prediction odor impressions [9]. These consist of a few thousand parameters, including constitutional descriptors, ring descriptors, topological indices, walk and path counts, connectivity indices, information indices, and 2D matrix-based descriptors. Sobel et al. predicted odor descriptors from 1513 physicochemical parameters using linear regression analysis based on the information extracted by principal component analysis [10]. The odor impression data they used were obtained from Dravnieks’s dataset [11]. The data consists of 146 odor descriptors and 180 odor samples. The prediction result for “Pleasantness” and “Edibility” is shown in Figure 1A,B. They reported that the first principal component of the physicochemical parameters had a positive correlation with odor pleasantness.

**Figure 2:**
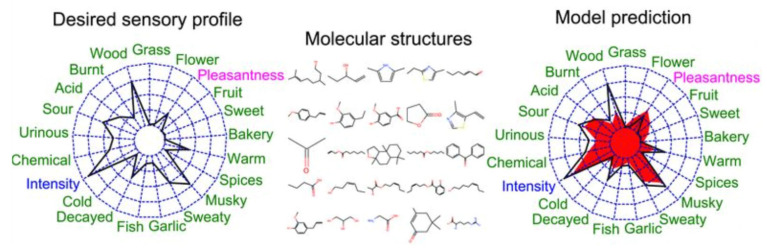
Prediction of odor-descriptor scores in DREAM project [12].

The other approach is known as the “DREAM” project [12]. Several teams tried to predict the perceptual ratings of 19 odor descriptors. The sensory test data of 476 mono molecules were collected by Keller & Vosshall [13]. The spider plot of their result is shown in Figure 2. They reported that regularized linear regression models showed the best performance.

**Figure 3:**
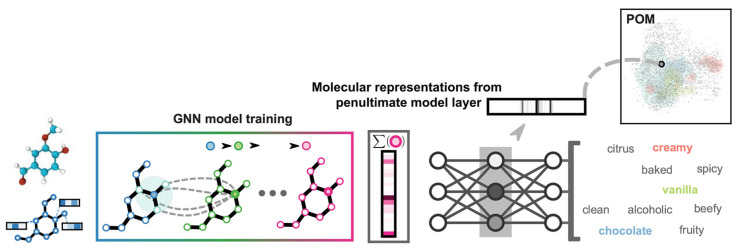
Principal odor map. From right to left, molecular graph representation of single molecules, Graph Neural Network, aggregation layer, Multi-Layer Perceptron and prediction. The intermediate layer is used as the odor map [16].

Shang et al. studied the odor-descriptor prediction using several machine leaning methods [14]. Meyer et al. converted the odor-descriptor ratings of the Dream dataset to Dravnieks’s one [15]. Moreover, Sanchez-Lengeling et al. proposed a principal odor map using a GNN (Graph Neural Network) to predict odor-descriptor scores as is illustrated in Figure 3 [16]. They used Good Scents [17] and Leffingwell & Associates’ dataset [18] including approximately 5000 molecules. They claimed that it predicted odor descriptors better than a median human panelist.

Although the works described above deal with mono molecules, it is indispensable to predict odor descriptors for mixtures. Although the work to predict odor descriptors for mixture is still limited, those studies are explained here.

**Figure 4:**
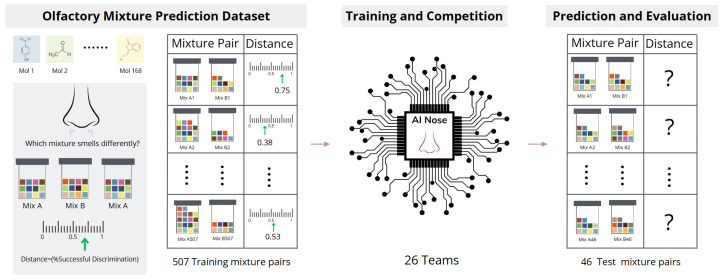
Overview of Dream olfactory mixture challenge, scheme of the data split [23].

Snitz et al. studied the perception similarity between mixtures and proposed a simple model [19]. Ravia et al. studied olfactory metamers based on that study [20]. Moreover, the prediction of the mixture has been attempted only recently [21,22]. Based on those works, a Dream olfactory mixture prediction challenge was organized as is shown in Figure 4 [23]. They used 507 mixtures for training. They evaluated RSME (Root Mean Square Error) and Pearson correlation coefficient. They claimed that the ensemble method combining several models showed the best performance. In addition to those works using molecular structure parameters, Hayashi et al. used an olfactory bulb map [24]. Although those works have been performed, it is still an open question to generalize to completely novel molecules.

#### 2.1.2. Odor-Descriptor Prediction Using Sensing Data

In the Section 2.1.1, the prediction methods from molecular structure parameters were described. However, sensing data rather than molecular parameters are necessary when combined with an odor reproduction technique, which enables the use of an odor-component set in an olfactory display (See Section 2.4).

Thus, the odor-descriptor prediction from chemical sensing data has been reported. Although a chemical sensing system called e-nose is often used to classify smells, the odor-descriptor prediction is more difficult. The examples of predicting odor descriptors using e-nose are described first.

**Figure 5:**
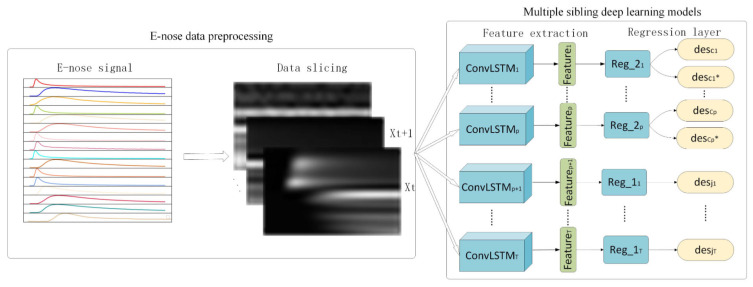
The overall system of the odor-descriptor prediction using QCM and metal oxide sensors. The experiment is mainly divided into two parts: (1) E-nose data preprocessing. It performs slice preprocessing on the electronic nose data based on the time series. (2) Multiple sibling deep learning models. Each sibling deep learning model has almost the same network structure consisting of convolutional LSTM layers and regression layers, except some have two outputs for jointly predicting paired descriptors and others only have one output [27]. An asterisk (*) after a descriptor denotes the descriptor most similar to the corresponding descriptor. Copyright IEEE 2021 with permission.

Yokoyama et al. studied odor-descriptor prediction from eight QCM (Quartz Crystal Microbalance, 10 MHz AT-cut) sensors using linear regression [25]. Hanaki et al. performed that prediction using QCM sensor array and a neural network called FLVQ (Fuzzy Learning Vector Quantization) [26]. Just recently, Guo et al. studied odor-descriptor prediction using QCM and metal oxide sensors. The sensor-array output pattern was mapped onto the odor descriptors using convolutional LSTM (Long Short-Term Memory) as is shown in Figure 5 [27]. However, it is not easy to generalize the odor-descriptor prediction since the information obtained from sensors is not always sufficient.

**Figure 6:**
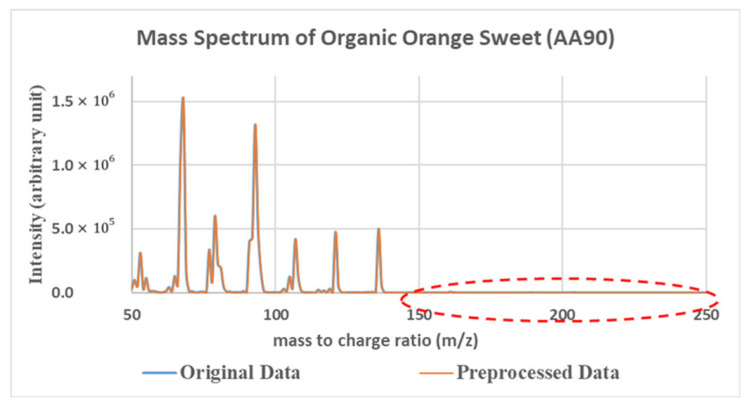
Example of mass spectrum (orange sweet). Noise in the red circle was removed after data preprocessing.

Another approach to obtain the sensing data is to use mass spectrometry [28]. Mass spectra are highly stable and contain rich information. Their dimension is typically a few hundred, which is much larger than a typical e-nose. An example of a mass spectrum is shown in Figure 6. Moreover, linear superposition is valid in a mass spectrum. Thus, we can calculate the mass spectra of mixtures without performing additional experiments.

**Figure 7:**
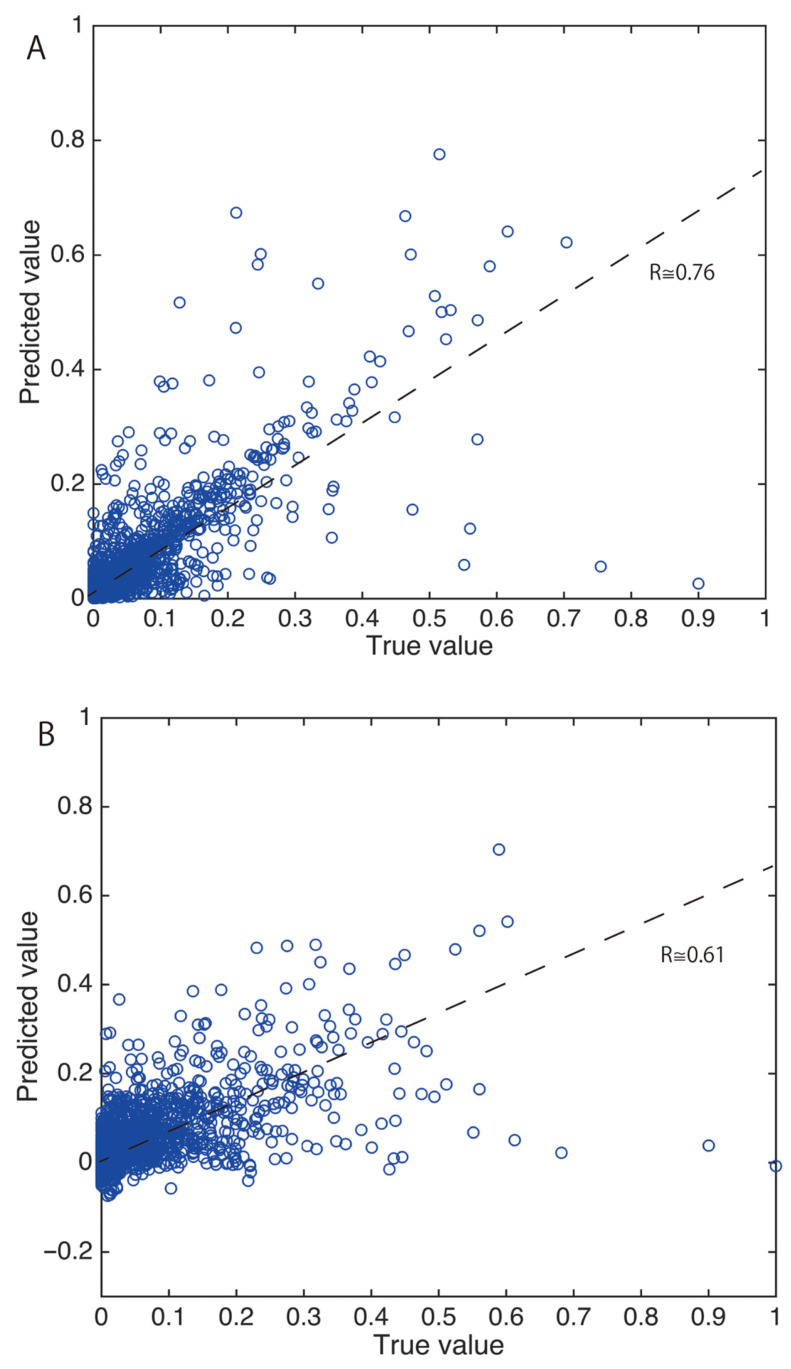
Prediction of odor-descriptor scores; (**A**) deep neural net with two auto encoders and (**B**) PLS method [31].

Although an SOM (Self-Organizing Map) [29] was used for the initial version of olfactory perception prediction from a mass spectrum [30], DNNs (Deep Neural Networks) were used later [31]. Two five-layer auto encoders [32], one for mass spectra and the other for sensory data were used to extract the features. An additional five-layer MLP (Multi-Layer Perceptron), made up of an input layer, three hidden layers and output layers, was used to map the mass spectrum feature onto the sensory one. Dravnieks’s dataset was used and a five-fold cross validation was performed. Cross validation is a method to evaluate pattern recognition capability when the data for evaluation is different from the training data. Its result is shown in Figure 7A. Moreover, a PLS (Partial Least Squares) method [33] was used to compare between them, shown in Figure 7B. PLS is a linear regression method with better prediction accuracy than a multiple linear regression. The number of latent variables was optimized in advance. In both figures, the prediction accuracy is good if many points are located close to the diagonal line. In the DNN, the correlation coefficient between ground truth and the prediction is around 0.76, whereas it is around 0.61 in the PLS. Thus, the prediction accuracy of the DNN is better than that of the PLS owing to its nonlinearity. However, the number of training data is not sufficient for further improvement in accuracy. Although it is difficult to increase the sensory data, it is possible to increase the number of mass spectra using a mass spectrum database [34]. Therefore, the accuracy was somewhat improved using that method [35]. Moreover, the odor-descriptor prediction for mixture was also confirmed [36].

**Figure 8:**
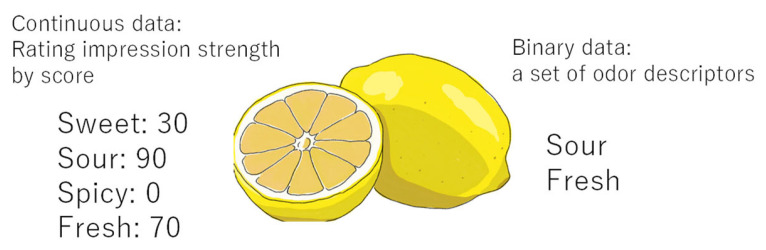
Difference between binary and continuous information.

However, a larger dataset including mass spectra and its corresponding sensory data can further improve that accuracy. Thus, organoleptic-evaluation data from chemical catalogs were used [37]. Although the amount of the data is much larger than Dravnieks’s dataset, only sensory binary data are available. Binary data represents a set of odor descriptors without the information on how well each odor descriptor matches the scent or its intensity, whereas Dravnieks’s data has continuous value in spite of its small dataset as is shown in Figure 8.

In the case of a binary dataset, the correlation between similar odor descriptors disappears, since only one odor descriptor among several similar ones appears in a mutually exclusive manner. Although the neural network may have difficulty in learning, clusters of similar odor descriptors can be formed using natural language processing techniques [38]. Word2vec, one of the typical natural language processing techniques, was used to obtain word embeddings, followed by clustering analysis to group similar odor descriptors [39].

**Figure 9:**
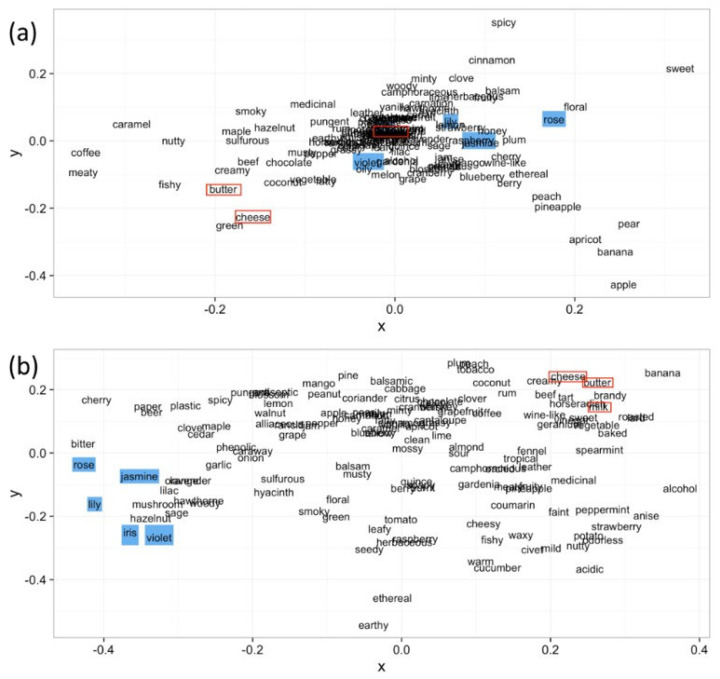
MDS diagrams based on correlation distance (**a**) and cosine distance obtained after NLP (**b**), the blue and red boxes show descriptors belonging to the same family [38].

To observe the word embeddings obtained from word2vec, the cosine similarity between them was calculated, and then MDS (Multi-Dimensional Scaling) [40] was performed to visualize the odor-descriptor map, as shown in Figure 9b. MDS is a technique to transform the distance matrix to coordinate information. For comparison, a correlation-based map is shown in Figure 9a. It was confirmed that the calculated similarity between descriptors differed significantly between the two methods. In the map obtained from word2vec, descriptors such as “Rose”, “Jasmine”, “Lily”, “Iris” and “Violet” are placed closer. That group is expected to be formed on the basis of “flower” impression. Moreover, “Milk”, “Cheese” and “Butter” are placed close to each other. The group is expected to be formed on the basis of “dairy product”. On the other hand, those descriptors are scattered in the correlation-based map as is shown in Figure 9a. Therefore, we can group odor descriptors based on their semantic meanings using word2vec, whereas just the correlation does not work. The accuracy of odor-descriptor prediction increased when grouping at the appropriate level was performed [38].

Odor-descriptor prediction has been studied, and now that prediction has become possible to some degree. The next step is the inverse method, i.e., sensing-data prediction from a set of odor descriptors.

### 2.2. Prediction Method of Sensing Data from Odor Perception

**Figure 10:**
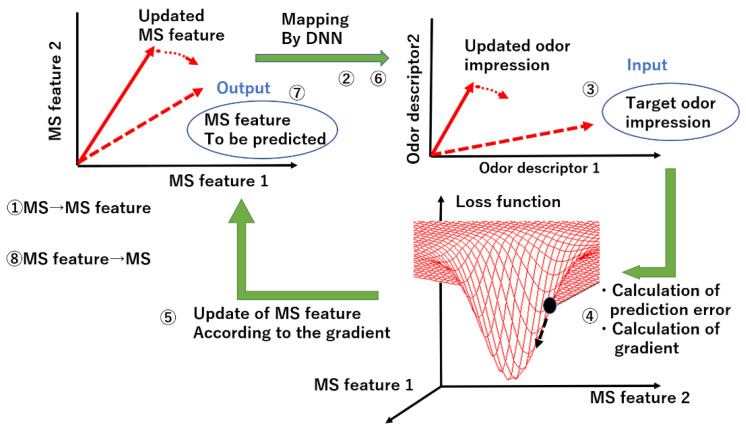
Method to predict mass spectrum from odor impression [41].

Although there have been many reports on odor-descriptor prediction from physicochemical parameters such as Dragon descriptors, the sensing-data prediction from a set of odor descriptors have not been studied well. This prediction enables the creation of an intended scent, since the sensing data can be decomposed into those of the ingredients. In this section, a computer simulation to predict the sensing data is presented [41]. Although its validation has been conducted only computationally, without experimental verification, this is the first step to automatic scent creation,

The sensing data used here are again mass spectra, because of their linear superposition property which allows mixtures to be handled without physical experiments. The method below is to solve this inverse problem. The overall algorithm is illustrated in Figure 10.

A DNN (five-layer MLP) to predict odor impression from mass spectrum is trained in advance using the DREAM dataset [12]. (1) First, the mass spectrum is mapped onto lower-dimensional data, i.e., MS feature, using an auto encoder. (2) Then, MS (mass spectrum) feature is mapped onto perceptual data by the DNN. (3) Thereafter, it is compared with the target odor impression and (4, 5) the MS feature is updated according to the gradient in the MS feature space on the basis of the loss function. (6) The updated MS feature is input to the DNN again, and the same procedure is repeated until an MS feature with minimum loss is found. (7, 8) Finally, that MS feature is converted back to MS again as the output using the auto encoder. The procedure to obtain the gradient analytically is described in Ref. [41].

**Figure 11:**
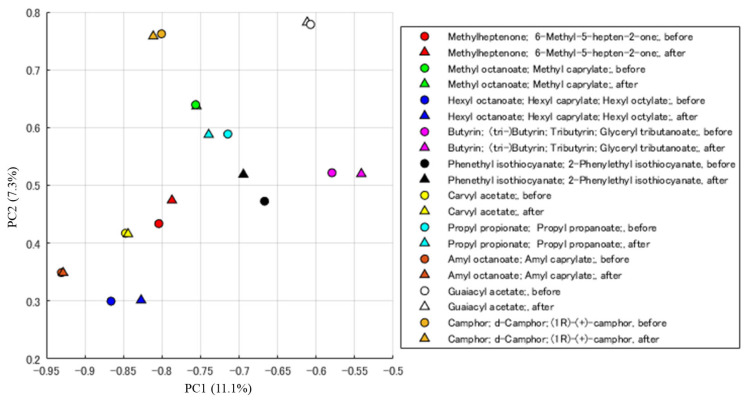
A PCA diagram of the MS feature before and after applying the gradient descent algorithm to 10 molecules [41].

Next, the gradient descent algorithm which iteratively reduces loss function according to the loss-function gradient, was applied to ten molecules contained in the DREAM dataset to predict the mass spectrum from the target odor impression. Its result is shown in Figure 11. The DNN prediction accuracy is not perfect as it contains some prediction errors in the odor-descriptor space. However, the location of each molecule in the PCA (Principal Component Analysis) diagram is almost the same after updating the MS feature according to the gradient descent algorithm. This means that the algorithm for searching the MS feature space works well. Moreover, another approach using SOM was also studied [42]. Since this is only the computer experiment, actual scent generation is explained in Section 3.

### 2.3. Odor Reproduction Using Odor Components

If the sensing data of the intended scent can be predicted, the next step is to establish the method to obtain the corresponding scent. It is preferable to approximate the scent by blending a small set of basic scents.

**Figure 12:**
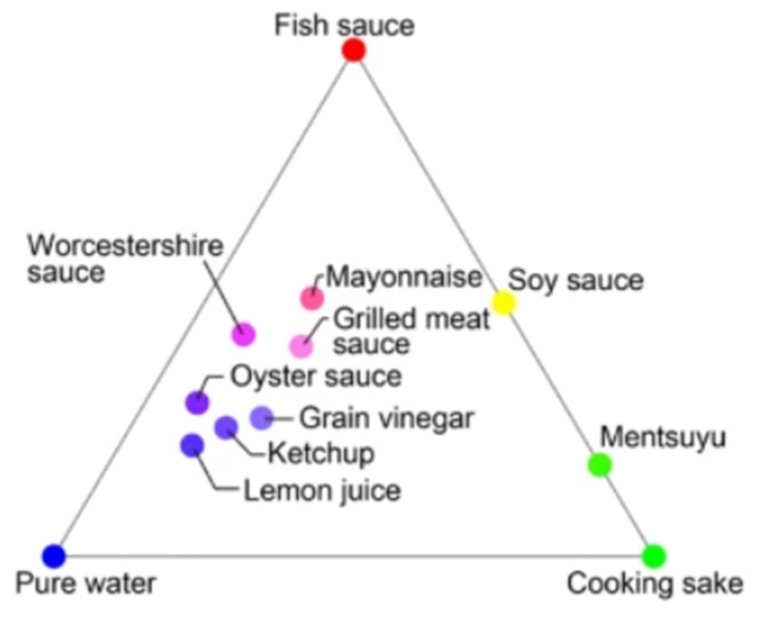
Color map of pure water and 11 seasonings. Fish sauce, cooking sake, and pure water are representative odors [43].

The representative scents called quasi-primary ones were studied using the sensing data [43]. Twelve MSS (Membrane-type Surface Stress) sensors were used. Three representative samples (extreme odors) within the collected dataset (twelve samples) were selected using an endpoint detection technique as is shown in Figure 12. It was found that the scents of the samples except representative ones were generated by blending the representative ones.

Although this is a simple example using the sensors, we can reproduce many scents using a small set of odor components, enabling us to make a scent corresponding to the mass spectrum obtained by the technique in Section 2.2. Each odor component acts as a basis vector so that a set of basis vectors can cover a large data space. Although a mass spectrum corresponding to the odor impression can be obtained, many chemicals are normally required to synthesize that mass spectrum. Thus, a small set of odor components to approximate the target mass spectrum is indispensable [44,45].

**Figure 13:**
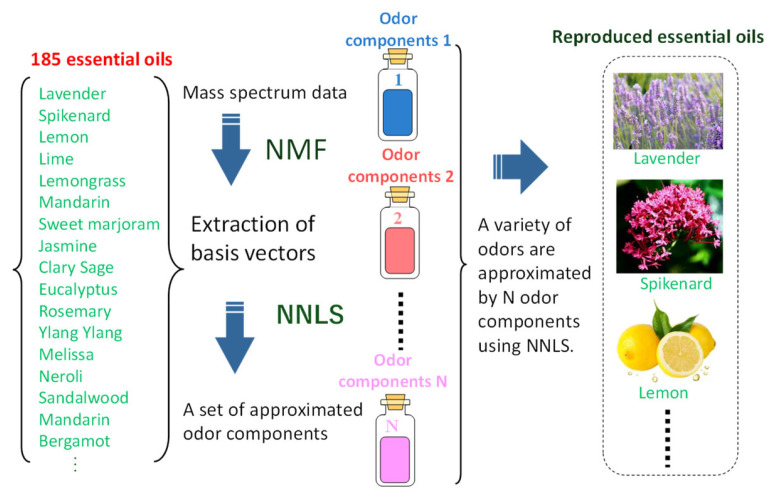
Procedure to find odor components for odor reproduction.

The procedure of odor reproduction is shown in Figure 13. Many samples are measured using mass spectrometry, although earlier work used QCM sensors in a system called “odor recorder” [46]. The data dimension of mass spectrum is about two hundred. Then, NMF (non-negative matrix factorization) [47] can extract a set of basis vectors that cover the whole set of mass spectra. NNLS (non-negative least squares) approximates each basis vector using the mass spectra of existing samples to obtain the mixture composition of each odor component. After obtaining mass spectra of odor components, NNLS is used to find the odor-component recipe of the target mass spectrum.

**Figure 14:**
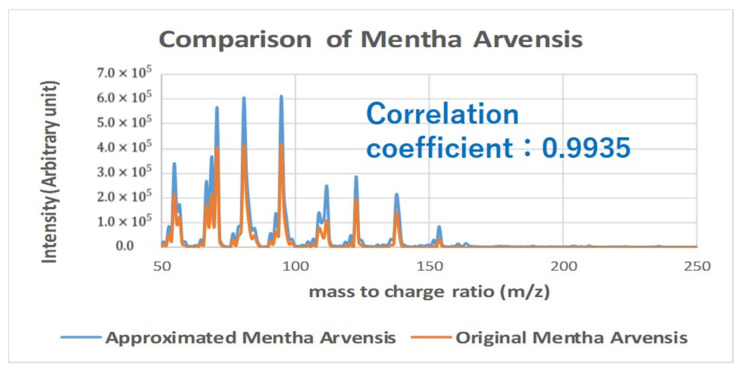
Approximated mass spectrum using odor components.

A mass spectrum is useful since it has the property of linear superposition so that a mass spectrum of mixture can be calculated as a weighted sum without actual measurement of the mixture. Example of a reproduced mass spectrum is shown in Figure 14. In total, 20 odor components synthesized from 185 essential oils were used for the odor reproduction. The approximated mass spectrum almost agrees with the original one (sample: mentha arvensis).

Moreover, the divergence between the original MS and the approximated one used for odor-component calculation was investigated. The divergence is a metric to measure the difference between mass spectra. Normally, the Euclidean distance or Kullback–Leibler (KL) divergence is used. However, the small-peak difference tends to be ignored in most cases. Although peaks at high *m*/*z* region are small, they often influence the olfactory perception. Thus, the small-peak difference is also important. Therefore, an Itakura–Saito (IS) divergence was used to solve the problem [48,49]. The sensory test revealed that the odor approximation by an Itakura–Saito divergence was better than the approximation by a KL divergence.

**Figure 15:**
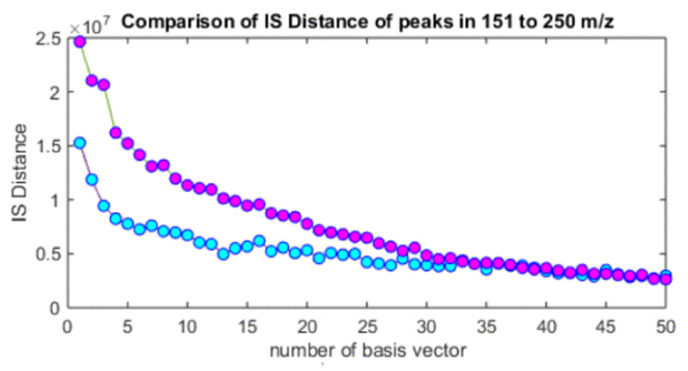
Dependency of residual error upon numbers of basis vectors for KL (purple dots) and IS (blue dots) divergences. Reproduced with permission from Ref. [50]. Copyright IEEE 2019 with permission.

Furthermore, the dependency of number of odor components upon the divergence was studied as is shown in Figure 15 [50]. The residual increases as the number of odor components decrease. However, the residual increase in IS divergence was smaller than in KL divergence. Therefore, IS divergence works well when the number of odor components is small. However, NMF based on IS divergence is sometimes instable. Many trials should be done to select the best one when IS-based NMF is used.

### 2.4. Olfactory Display Using Odor Components

Next, odor reproduction using an olfactory display is explained. Although several types of olfactory displays have been reported [51,52,53], the olfactory display to blend many ingredients precisely was not reported previously. However, this function is indispensable for odor reproduction described in the Section 2.3.

**Figure 16:**
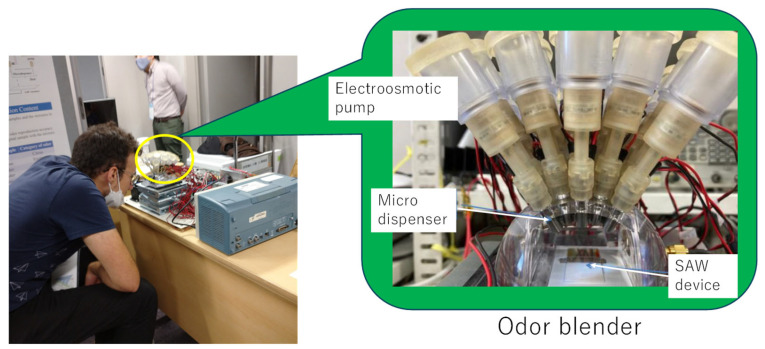
Photo of olfactory display to reproduce odors.

Recently, the multi-channel olfactory display based on a SAW (Surface Acoustic Wave) atomizer [54] was developed [55,56]. A photo of the olfactory display is shown in Figure 16. Up to 20 ingredients can be blended with an arbitrary ratio using this olfactory display. Each ingredient is stored in an independent channel and supplied by a micro-dispenser. Nano litter-size droplets ejected by micro dispensers are atomized by the SAW atomizer owing to the acoustic streaming effect. The generated mist is then mixed and presented as an odor. Since each channel can be controlled independently, the ratio of each ingredient can be adjusted flexibly according to the calculated recipe. Therefore, the odor can be immediately reproduced if the odor component derived from the method in Figure 13 is set at each channel. The odor reproduction of essential oils was experimentally confirmed by the sensory test, i.e., triangle test [56,57]. In the test, seven essential oils were examined using the 20-component olfactory display with 40 subjects. The results showed that it was difficult to distinguish between the reproduced odor and the target odor, and no significant difference was observed at the 5% significance level for all tested essential oils. These results suggest that odor reproduction by the olfactory display was basically achieved, although odor features, intensity, and wind difference were sometimes used as clues for discrimination.

**Figure 17:**
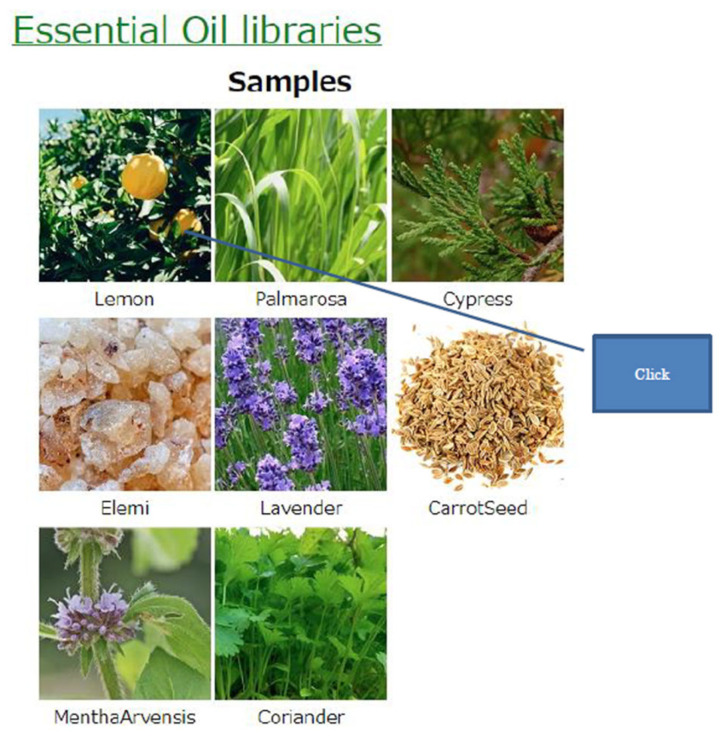
Web site called 100 selections of essential oils.

Then, the web site called 100 selections of essential oil was developed as is shown in Figure 17. A user can select the essential oil on the screen, followed by the immediate presentation of the selected scent by the olfactory display [58]. The user is first requested to select one of seven essential oil categories [59] and then, to select the desired essential oil. The selected odor is reproduced by the olfactory display using the odor components derived from essential oils by non-negative matrix factorization (NMF) and a non-negative least squares method as described above. The odor reproduction technique is one of the fundamental techniques to realize an AI-based scent creation system.

## 3. Optimization Technique for Scent Creation

**Figure 18:**
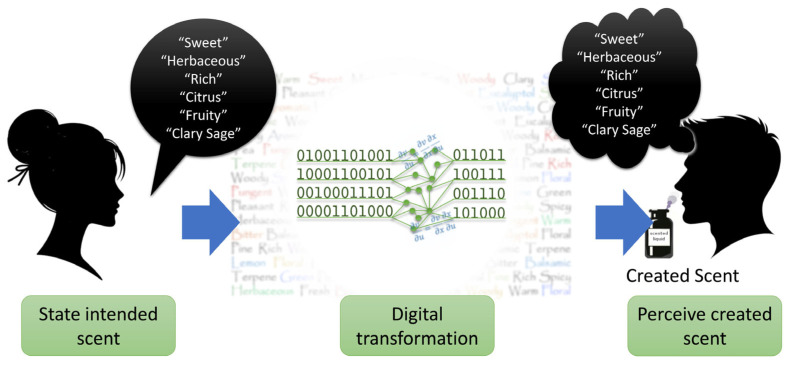
Conceptual overview of the generative scent creation process. A user states an intended scent, specifying the intended odor descriptors (**left**), which are processed through a digital transformation stage based on a generative process (**center**). The system then produces a physical scent corresponding to the specified descriptors, which is subsequently perceived by the user (**right**). Taken from [60].

Automatic scent creation represents a critical step beyond odor prediction and odor reproduction, enabling the generation of new scents corresponding to predefined odor impressions. However, previous approaches have been validated only computationally, and experimental validation through human sensory evaluation is required to confirm that the generated scents can be physically realized and perceived as intended. To address this limitation, Aleixandre et al. [60] conducted the first experimental validation of the framework as described in this section. Essential oil data were employed because they can be safely blended and evaluated by human participants, enabling the physical realization of the algorithmically generated mixtures. This approach allowed validation of the complete scent creation pipeline as illustrated in Figure 18. The primary novelty of this work lies in the physical realization of the smells and the integration of sensory experimental validation within the generative procedure itself.

**Figure 19:**
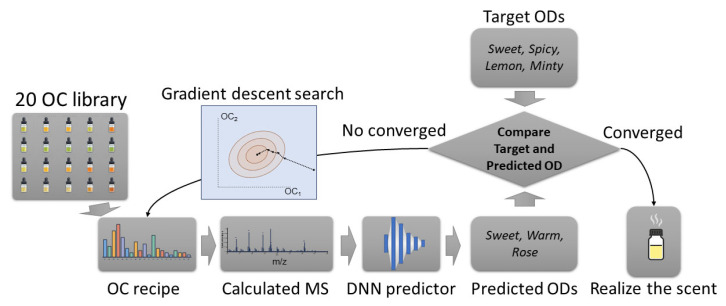
Scheme of the automatic scent creation workflow. The workflow begins with a library of 20 odor components, illustrated as 20 vials. A candidate mixture is defined as an OC (odor-component) recipe, represented here as a bar plot of the relative proportions of the odor components. From this recipe, a mass spectrum is calculated and used as input to a DNN that predicts the associated odor descriptors (OD). These predicted descriptors are compared with the target descriptor set, and the mismatch is iteratively reduced by gradient descent, which updates the component ratios. The final optimized recipe is then physically realized as the resulting scent. Adapted from [60].

Following the scent generation methodology described in the previous sections, this automatic scent creation system consisted of three main components: an odor-descriptor prediction model, an odor-component representation, and an optimization algorithm that connects perceptual targets with scent mixtures. The optimization is to find a set of variables to minimize a cost function. Figure 19 shows a detailed view of the algorithm. A recipe is first defined using the odor-components, represented as a vector of component ratios. From this recipe, a mass spectrum (MS) is calculated and used as input for the DNN that predicts the corresponding odor descriptors. The predicted descriptors are compared with the target descriptors, and the resulting error is minimized by iteratively updating the odor component ratios via gradient descent. The gradient of the error with respect to each component ratio was derived analytically, allowing direct computation of the update direction at each iteration. Once the predicted and target odor descriptors are brought into sufficient agreement, the optimized recipe is used to realize the final scent as a physical mixture.

The development and evaluation of the system were based on a dataset consisting of 180 commercially available essential oils. For each essential oil, mass spectra were measured, and odor-descriptor information was compiled for 94 of them. The mass spectra consisted of 201 intensity values corresponding to different mass-to-charge ratios from 50 *m*/*z* to 250 *m*/*z*. Mass spectrometry measurements were performed using electron ionization, and each essential oil was measured multiple times to improve signal stability and reduce noise. The odor-descriptor data consisted of 39 binary variables indicating the presence or absence of specific perceptual descriptors. Binary odor descriptors were used because such data are commonly available in fragrance databases. Although binary descriptors provide less detailed information than continuous ratings, such as the DREAM database in Section 2.1.1, they are sufficient to represent the presence or absence of key perceptual features and enable the development of prediction and synthesis algorithms. The data are available openly in [41,60].

**Figure 20:**
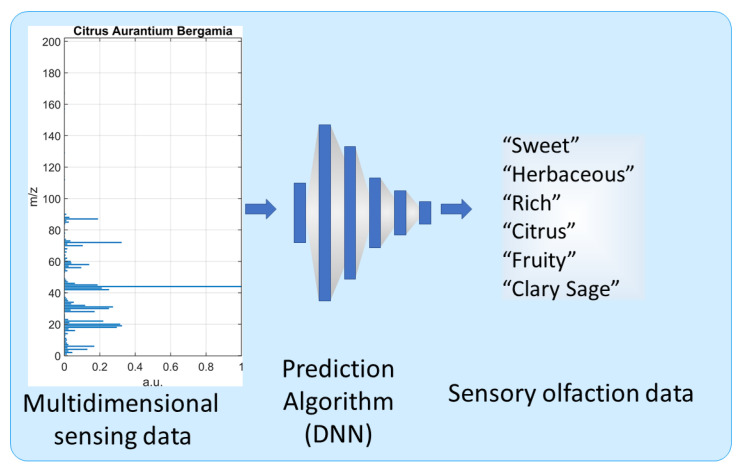
Conceptual diagram of the deep neural network employed in the generative pipeline. The model maps (MS measurements (**left**) to corresponding sensory odor descriptors (**right**)), enabling prediction of odor impressions from analytical sensing data. Adapted from [60].

Using a dataset of 94 essential oils, each associated with a mass spectrum and sensory descriptors, an odor-descriptor prediction model was developed to link sensing data with perceptual information (Figure 20). The model takes the 201-dimensional mass spectrum as input and predicts 39 odor descriptors as output. Because a single scent can be described by several odor descriptors at the same time, the model was designed for multi-label prediction. To learn the relationship between the measured spectra and the perceptual descriptors, a DNN with several intermediate layers was used. The model was trained to make its predictions as close as possible to the known descriptors, while including regularization to improve robustness and reduce overfitting. Overfitting is a situation where the model fails in generalization for new, unseen data even if it is trained by the detailed data.

Because the number of training samples was limited compared with the large number of network parameters, regularization techniques played an essential role in preventing overfitting, reducing co-adaptation and effectively lowering model capacity. In particular, dropout regularization was applied to the deeper hidden layers and regularization was included in the loss function to encourage sparsity in the network weights. These regularization techniques were critical to stabilize the training process and improve the predictive performance of the model. The performance was evaluated using leave-one-out cross-validation, in which one sample was used for testing while the remaining samples were used for training, and this procedure was repeated for all the 94 samples.

The overall balanced accuracy of the model was 0.736, indicating that the network was able to capture the relationship between sensing data and perceptual descriptors for the essential oils with good accuracy. However, prediction performance varied among descriptors. Some descriptors, such as floral-related descriptors, showed higher prediction accuracy, while others, like “sweet” and “fresh”, showed lower performance. This variation has important implications for scent creation, as errors in descriptor prediction can affect the accuracy of the generated scent mixtures. Despite these limitations, the prediction model provided sufficiently accurate mapping to enable the algorithmic design and scent synthesis.

The trained neural network provided a mapping with a differentiable function from mass spectrum space to odor-descriptor space. This property was essential for the scent creation process, because it enabled calculation of gradients with respect to the sensing data, which were used in optimization to determine the odor-component mixture corresponding to a target perceptual descriptor set.

The next building block of the method was the odor-component representation, which provided a practical way to synthesize scents. Instead of directly manipulating individual essential oils, the system represented scents as mixtures of a limited number of odor components derived using non-negative matrix factorization. As described in Section 2.3 odor components were derived using non-negative matrix factorization applied to mass spectra from the 180 essential oils. A total of 20 odor components were obtained.

Then, the optimization algorithm performed inverse mapping from perceptual descriptors to physically realizable odor-component mixtures based on the essential oils. Starting from an initial mixture defined in the odor-component space, the corresponding mass spectrum was calculated using the linear superposition property of the components. This mass spectrum was then used as input to the neural network to predict the associated odor-descriptor set. The difference between the predicted descriptors and the target descriptors was defined as the loss function, and the odor-component ratios were iteratively adjusted using gradient descent to minimize this loss. Because the mapping from odor-component ratios to mass spectrum and subsequently to odor-descriptors is differentiable, the gradient of the loss function with respect to the odor-component ratios can be calculated using the chain rule, allowing direct optimization of the mixture composition.

A key innovation of this approach was the integration of an odor-component representation into a gradient-based optimization framework. In previous inverse design methods, optimization was performed directly in the sensing-data space, i.e., in the mass spectrum space, which can produce solutions that are difficult to realize with a limited number of odor components [41]. In contrast, the present method constrained the search to the odor-component ratio space. At each iteration, the current recipe was converted into a mass spectrum by linearly combining the odor-component spectra, and this spectrum was then evaluated by the odor-descriptor prediction model. The difference between predicted and target odor descriptors was used to update only the odor-component ratios. This constrained gradient search made it possible to identify mixtures predicted to produce the desired odor impression while remaining compatible with the physical synthesis model.

The validity of the scent creation method was evaluated through sensory experiments involving human subjects [57]. This experimental validation was essential because computational agreement between predicted and target descriptors does not guarantee perceptual equivalence. The sensory tests were designed to evaluate whether the algorithm could modify a scent in a predictable way by adding a specific odor-descriptor. First, an existing essential oil was selected as the reference scent. The corresponding odor descriptor set was then modified by adding one additional descriptor. The algorithm was used to generate a new odor-component mixture predicted to correspond to this modified descriptor set.

Both the original and modified mixtures were physically prepared by blending odor components according to the calculated recipes. The samples were diluted to ensure comparable intensity and presented to participants in a double-blind procedure. A total of 23 nonprofessional panelists took part in the experiments. In each trial, participants were presented with two samples and asked to identify which sample exhibited the added odor descriptor more strongly. The experiments were repeated using different base scents and descriptors. This comparative evaluation approach was adopted instead of absolute descriptor rating, such as selecting all applicable descriptors, because comparative judgments are easier and more reliable for non-professional panelists [57]. Absolute rating requires participants to independently assess the presence and intensity of multiple descriptors, which is a more complex task and may lead to higher variability. In contrast, comparing two samples with a known difference allows participants to focus on relative perceptual changes, providing a more robust method to evaluate whether the intended descriptor modification was successfully achieved.

**Figure 21:**
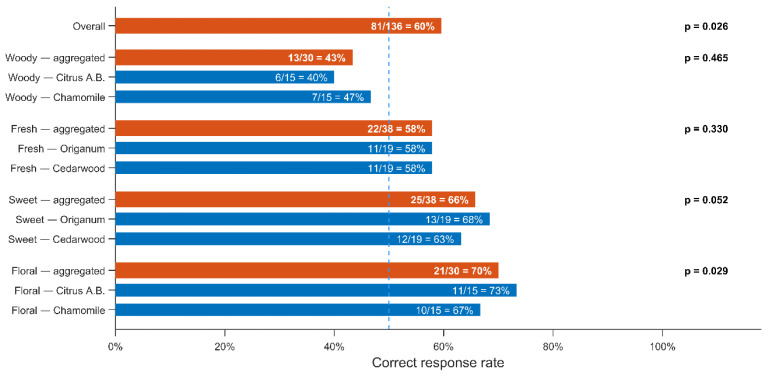
Correct response rates in the sensory evaluation tests. The figure shows the proportion of correct responses for each odor-descriptor condition (blue bars), along with the aggregated results across sensory tests (orange bars). The dashed line indicates the 50% chance level. Adapted from [60].

As shown in Figure 21, the results showed that participants were able to identify the modified scents at rates exceeding the chance level expected for a binary choice task. When all trials were considered together, the overall correct identification rate was significantly higher than 50%, indicating that the descriptor modification introduced by the algorithm produced perceptible differences. Statistical significance was evaluated using a binomial test [57], confirming that the observed success rate was unlikely to occur by random selection.

This variation is consistent with the prediction performance of the neural network model. Descriptors that were predicted more accurately resulted in more reliable scent modifications, whereas descriptors with lower prediction accuracy led to less perceptible changes. These results indicate that the effectiveness of the scent creation algorithm is directly influenced by the accuracy of the odor-descriptor prediction model.

These results demonstrate that the proposed method is capable of generating scent mixtures with perceptually meaningful and predictable modifications, as confirmed by statistically significant sensory test outcomes. The experiments showed that descriptor-guided scent design is achievable and can be experimentally validated, while also revealing that the effectiveness of the scent creation algorithm is directly influenced by the accuracy of the odor-descriptor prediction model. In particular, descriptors predicted with higher accuracy resulted in more reliable perceptual modifications, highlighting the importance of the prediction stage within the overall framework. Although the current system is limited by the size of the available dataset and the prediction accuracy of the neural network, the integration of descriptor prediction, optimization, and odor reproduction provides a feasible approach for automatic scent creation. Further improvements in dataset size, descriptor representation, and prediction performance are expected to enhance the reliability and precision of the generated scents.

## 4. Generative AI for Scent Creation

Automatic scent creation can also be achieved using generative models that directly produce sensing data corresponding to specified odor descriptors. In contrast to optimization approaches that adjust existing spectra, generative diffusion networks learn the probability distribution of mass spectra conditioned on perceptual descriptors and can generate new spectra. A diffusion model is a type of generative AI that creates data by gradually adding noise to it and then learning to reverse this process to generate new, high-quality outputs.

The core of the method described in this section is a generative diffusion network [61], referred to as OGDiffusion [62]. It was specifically designed to generate mass spectra corresponding to predefined odor descriptors using essential oils as the synthesis basis. The generated mass spectra were subsequently converted into physically realizable mixtures using non-negative least squares, allowing experimental validation through sensory evaluation. This approach demonstrated that generative models can also produce scent recipes aligned with perceptual targets and physically realizable using essential oils.

The system was developed using the same dataset of essential oils for which both mass spectra and odor-descriptor information were available. The mass spectrum consisted of the same 201 intensity values corresponding to different mass-to-charge ratios. Odor-descriptor information was improved by collecting additional information for diverse fragrance databases and manufacturer sources. After processing, 57 odor descriptors were identified across 166 essential oils. To ensure sufficient representation for training, only the nine most frequent descriptors were selected, each appearing in more than 20 essential oils. These descriptors are: fresh, wood, herbal, floral, spicy, green, warm, sweet, and balsamic. The data are available in [62].

**Figure 22:**
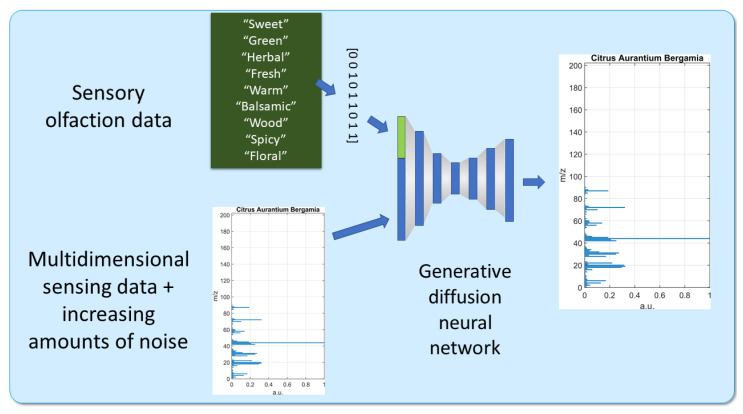
Schematic representation of the OGDiffusion framework. MS data with progressively increasing noise are combined with binary odor-descriptor inputs and processed by the generative diffusion neural network. The model learns to reconstruct clean spectra during training and, at inference, generates new mass spectra conditioned on specified perceptual descriptors. Adapted from [62].

The scent generation model was implemented as a diffusion-based denoising autoencoder that learns to reconstruct mass spectra from noisy inputs while being guided by odor-descriptor information (Figure 22). The input to the model combined the mass spectrum of an essential oil with its odor-descriptor vector, and Gaussian noise was added to the spectrum during training. The model was then trained to recover the original clean mass spectrum, allowing it to learn the relationship between perceptual odor descriptors and MS sensing data. The network was built with several fully connected layers, including a narrower intermediate layer that compressed the information before reconstruction, and the final output was a 201-dimensional mass spectrum. To improve robustness and reduce overfitting, dropout regularization was applied and the amount of Gaussian noise was gradually increased during training. This strategy helped the model learn more stable and reliable mappings, so that it could recover plausible spectra even from strongly corrupted inputs.

During inference, the input consisted of random Gaussian noise instead of the MS spectral lines and a desired odor-descriptor binary vector. The network then generated a mass spectrum at the output that corresponded to the specified descriptors, effectively performing conditional generation of sensing data.

Before sensory validation, several analytical tests were carried out to assess the quality of the generated mass spectra. First, the model was tested on its ability to reconstruct the original spectra of essential oils from noisy inputs, showing high agreement with the measured spectra. Second, the generated spectra were examined to determine whether they were consistent with the target odor descriptors, and the results indicated that the generated outputs tended to correspond to oils with similar perceptual profiles. Third, an independent prediction model was applied to the generated spectra and was able to recover descriptor information with performance comparable to that obtained for real essential oil spectra. Overall, these validations suggest that the model generated spectra that were both plausible and meaningfully related to the intended odor descriptors.

**Figure 23:**
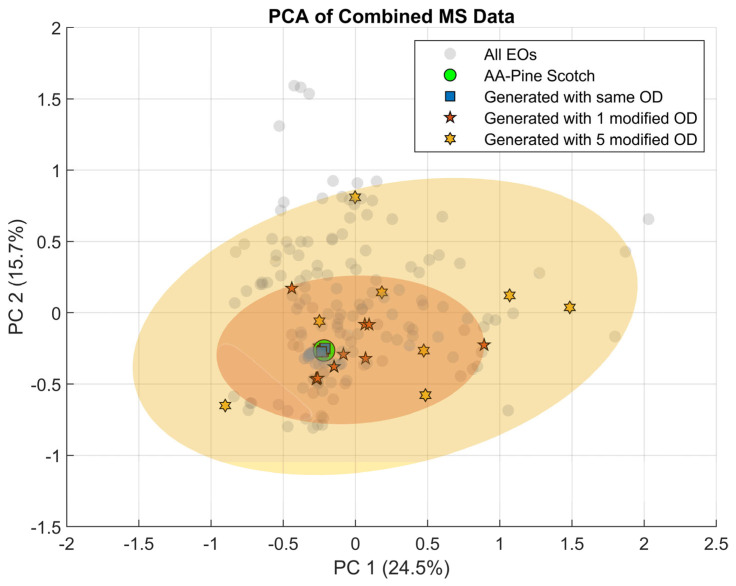
PCA representation of mass spectrometry data. Gray markers correspond to the complete set of essential oils (EOs). The original Pine Scotch essential oil is shown as a green marker. Spectra reconstructed by the OGDiffusion model under identical descriptor conditions are displayed as blue squares. Spectra generated after modifying one odor descriptor (OD) are indicated by orange star symbols, while those obtained after modifying five descriptors are shown as yellow star symbols. The shaded ellipses represent the 90% confidence regions, computed by assuming a Gaussian distribution over the nine generated samples in each condition. Taken from [62].

Repeated generations using the same odor-descriptor set but different random seeds produced spectra and recipes that were similar, although not identical. This indicates that the model can generate more than one plausible solution for the same target odor profile. For example, two generated recipes may differ slightly in the proportions of their odor components, while still corresponding to the same predicted descriptors. In contrast, when the target descriptor set was changed, the generated spectra also shifted in a consistent way in PCA space (Figure 23). This shows that the model was not varying randomly, but was responding meaningfully to the conditioning descriptors. Together, these results suggest that the model preserves meaningful variability while remaining guided by the target odor profile.

The final validation of the scent creation framework was conducted through human sensory evaluation using physically realized mixtures. To enable this validation, the mass spectra generated by the OGDiffusion network were converted into essential oil formulations. This conversion was performed using non-negative least squares (NNLS), which approximates each generated mass spectrum as a non-negative weighted combination of the mass spectra of the available essential oils. Through this reconstruction step, the generated spectral representation was translated into physically realizable recipes. The integration of conditional generative modeling with NNLS-based mixture reconstruction therefore enables a complete pipeline, from perceptual descriptor input to tangible scent creation.

The resulting essential oil mixtures were prepared in odor vials and evaluated using double-blind sensory procedures. Assessors were voluntary participants representing a general audience and did not receive specialized training. All mixtures were prepared in single batches to ensure consistency, and presentation order was randomized to minimize bias.

**Figure 24:**
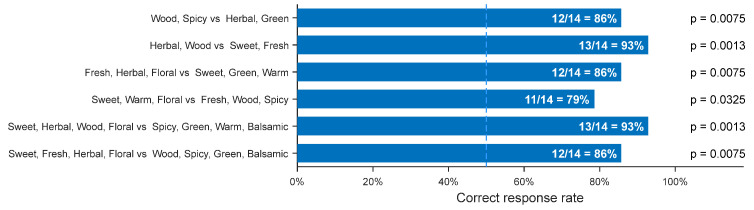
Correct classification rates for generated mixture pairs in sensory test. Adapted from [62].

Three sensory tests were conducted to evaluate perceptual validity. The first test assessed whether participants could reliably identify two generated mixtures corresponding to different odor-descriptor sets. The second test evaluated the perceptual presence of individual odor descriptors using a two-alternative forced choice (2-AFC) protocol, a test that allows objective evaluation with strong statistics. The third test examined whether the generated mixtures expressed specific odor descriptors more strongly than randomly selected natural essential oils lacking those descriptors. Figure 24 shows the result for the first test. Across all experiments, participants classified the mixtures at statistically significant rates (*p* < 0.05), indicating that descriptor-conditioned generation produced perceptually distinct aromas. Together, these sensory results demonstrate that the generated spectra are not only analytically coherent but also perceptually meaningful when realized as physical mixtures.

Additionally, to evaluate the potential automation of the procedure, complementary tests were conducted using a 20-channel olfactory display [63]. The system implemented the same OGDiffusion framework, but the generated spectra were realized using the set of 20 odor components introduced previously with NNLS. These components constituted the basis of the display system, allowing the generated spectra to be approximated and reproduced through controlled mixing of the scents in the available channels. The system enabled real-time blending and delivery of scent mixtures corresponding to the descriptor-conditioned outputs of the generative model.

**Figure 25:**
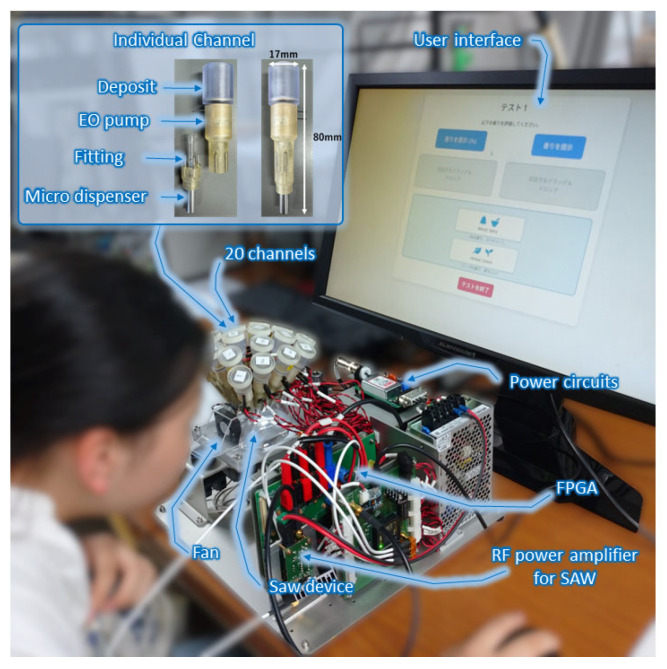
Experimental olfactory display system. It illustrates a participant operating the device, with principal hardware components identified. A magnified inset details a single odor channel, showing the liquid reservoir, electro-osmotic pump, and microdispenser responsible for controlled droplet release. Taken from [63] with permission.

A sensory evaluation was conducted with 26 voluntary participants during a public demonstration event at the Institute of Science Tokyo. The experimental setup, including details of the odor display, is illustrated in Figure 25. Participants performed a forced-choice classification task across six pairs of descriptor sets, identical to those used in the essential-oil-based validation. The aggregated accuracy across all questions was 62.2%, with a statistically significant binomial test result (*p* = 0.0015), indicating performance above chance level. The overall results confirm that descriptor-conditioned scent generation remains effective when implemented through a reduced 20-component basis and delivered via an atomized olfactory display.

These results demonstrate that the proposed generative framework is robust across multiple stages of validation, including analytical reconstruction, physical realization through essential oil mixtures, and operation within a hardware-constrained olfactory display system. Together, these outcomes establish a practical foundation for digital olfaction by enabling the automated transformation of perceptual odor descriptors into physically realizable scent mixtures. Although the current implementation is constrained by the limited dataset size and by the relatively small set of odor descriptors used during training, the results clearly demonstrate the feasibility of descriptor-driven scent generation. Expanding the descriptor space and increasing the volume and diversity of training data are expected to further improve prediction accuracy, robustness, and generative expressiveness. Such developments may enable future applications in fragrance design, immersive virtual environments, and human–computer interaction systems incorporating controlled olfactory output.

## 5. Discussion

Two types of scent creation techniques, an optimization technique and generative AI, were shown. The performances of both techniques seem to be comparable if both methods work smoothly. However, the optimization method uses a gradient, which is often sensitive to noise. It is necessary to suppress the noise influence as much as possible when we use a gradient in a real problem, which is different from just a simulation. Moreover, the parameters to update the odor-component recipe should be determined empirically. Experience in selecting these parameters is necessary, since their values change according to the situation. Methods such as Savitzky–Golay filtering pre-processing of the MS and adaptive learning-rate strategies may help reduce noise sensitivity and improve robustness in future developments.

On the other hand, we intentionally add noise to the signal for training the diffusion model. It can enhance robustness against the noise and unexpected signal changes for predicting a mass spectrum from a set of odor descriptors. From the viewpoint of robustness against noise and unexpected change in experimental conditions, the diffusion model seems better at the current stage. Although we focused on the diffusion model here, other AI techniques, e.g., GANs (Generative Adversarial Networks), VAEs (Variational Auto Encoders), and transformers, etc., can also be investigated and tested. Comparative studies across these architectures may clarify the trade-off between robustness, controllability, and diversity of generated scents.

Moreover, this work is not limited to scent creation alone. We also described odor-impression prediction, odor reproduction with odor components, and olfactory display. These elements can be regarded as components of a broader framework for digital scent technology. In this sense, the significance of the present work lies not only in the scent creation technique itself, but also in showing how these different techniques can be connected within one system.

Explainability remains an important open issue in AI-based scent creation. The original studies reviewed here mainly focused on predictive or generative performance, while more direct interpretability analyses have not been investigated in depth. Future work should include methods such as SHAP (SHapley Additive exPlanation), attention-based analysis, feature-importance evaluation, and latent-space analysis in order to better understand how odor descriptors and sensing data are related within the models.

The techniques used here are based on mass spectrum. The mass spectrum is used here because large-scale mass spectrum data are easily available and linear superposition theorem is valid. Especially, the latter property is very important since we can easily obtain the mass spectrum of the mixture without performing any experiment. Moreover, we focus on algorithmic development since mass spectrometry is an already established technique. However, it would be preferable to replace it with a sensor array because it is very expensive and bulky. The mass spectrometer here works in the same way as a sensor array. We expect that drastic progress of e-nose technology will change the situation greatly. Table 1 shows a summary of the two method characteristics.

Subjective variability cannot be avoided in scent creation. The variability occurs in absolute sensory evaluation. However, relative change in perception is more stable than absolute evaluation. We think we can make the intended scent when we repeatedly recreate the scent with reference to the previous scent.

A major limitation of the current field is the scarcity of large, standardized public datasets suitable for scent creation. The datasets often used in computational olfaction are summarized in reference [64]. Public resources such as DREAM, Dravnieks, and NIST are highly valuable and are discussed in this review, but they mainly concern mono-molecular perceptual data or reference spectra of individual compounds rather than descriptor-conditioned mixture generation with experimental validation. As a result, direct benchmarking across studies remains difficult, especially for physically realizable scent-creation tasks.

## 6. Conclusions

We described odor-impression prediction, odor reproduction, and olfactory display, followed by scent creation techniques. The technique to create scents automatically has just started being used. It was validated by this small-scale experiment at the current stage. We need more data, especially sensory data, to apply this technique to real problems. The collection of high-quality large-scale data is essential to enhance system capability. Then, AI-based scent creation will bring remarkable effects to various industries in the future.

## Figures and Tables

**Table 1:** Comparison of the characteristics of two methods.

Aspect	Optimization-Based Scent Creation	OGDiffusion-Based Scent Creation
Basic principle	The odor recipe is adjusted step by step so that the predicted descriptors become closer to the target descriptors	A diffusion model learns to generate mass spectra from odor descriptors
Inference input	Target odor descriptors	Target odor descriptors and random noise as generative seed
Main internal process	Gradient optimization updates the recipe through the prediction model	Denoising generates a mass spectrum guided by the target descriptors
Output before realization	Optimized odor-component ratios	Generated mass spectrum
Conversion to physical scent	Directly connected to odor components, so it can be realized immediately	Requires an additional NNLS step to convert the generated spectrum into a realizable recipe
Nature of solution	Usually gives one optimized solution for each target and starting point	Can generate several plausible solutions for the same target descriptors
Main strengths	Direct objective control, easy to include physical constraints, and clear connection to realizable mixtures	More flexible generation, can produce multiple plausible outputs, and is more robust to noise
Main limitations	Sensitive to noise, initialization, and empirical parameter tuning	Requires model training and sufficient data; explainability is limited

## Data Availability

Any new data were not used.
